# Pediatric Craniovertebral Junction Anomalies: A Literature Review

**DOI:** 10.7759/cureus.87164

**Published:** 2025-07-02

**Authors:** Quang Dai La, Nehal Revuri, Aiman Baloch, Shanmukh Bachhu, Muhammad Ayub, Sobia Ahmed, Sumalatha Aradhya, Noman Sadiq

**Affiliations:** 1 Surgery, The Innovative STEMagazine, College Station, USA; 2 Biology, Texas A&M University, College Station, USA; 3 Medicine, Mekran Medical College, Turbat, PAK; 4 Medicine, The Innovative STEMagazine, College Station, USA; 5 Civil Engineering, University of California, Berkeley, Berkeley, USA; 6 Civil Engineering, The Innovative STEMagazine, College Station, USA; 7 Radiology, Bolan Medical Complex Hospital, Quetta, PAK; 8 Computer Science and Engineering, Siddaganga Institute of Technology, Tumkur, IND; 9 Physiology, Mekran Medical College, Turbat, PAK

**Keywords:** craniovertebral junction anomalies, cvj, cvj anomalies, cvj malfunctions, pediatric craniovertebral anomalies, pediatric cvj

## Abstract

Pediatric craniovertebral junction (CVJ) abnormalities comprise a heterogeneous and multifaceted collection of congenital and CVJ malformations that involve the occipital bone, C1, and C2, as well as associated ligamentous structures. They have the potential to severely impair neurologic function through cervicomedullary junction compression, instability, or vascular insufficiency. Early diagnosis and urgent surgical intervention are crucial to optimizing neurological outcomes in children with this condition, but the anatomical and biomechanical features of the pediatric spine present special diagnostic and therapeutic challenges. Through an analysis of recent literature, we outline current diagnostic modalities and treatment strategies while emphasizing recent innovations in surgical planning and technique.

Chiari malformation type I (CM-I) is the most common CVJ abnormality in children and is characterized by the downward protrusion of the cerebellar tonsils below the foramen magnum. CM-I is generally found in children undergoing MRI for symptoms such as headache, neck pain, or neurological dysfunction, and syringomyelia in a minority of instances. Atlantoaxial instability, often encountered in children with Down syndrome, and Grisel’s syndrome, a non-traumatic atlantoaxial subluxation most commonly resulting from upper respiratory infections, are other common CVJ anomalies. They impact quality of life significantly and must be properly planned preoperatively to avoid the possibility of neurological injury.

Advances in technology, including imaging using MRI, CT, and dynamic imaging, have greatly improved diagnosis and surgical planning for CVJ anomalies in children. Intraoperative navigation systems provide real-time images to permit precise screw placement, reducing neurovascular complications and improving the success of surgery. Three-dimensional printing technology allows for patient-specific surgical planning with enhanced anatomical visualization and individualized treatment plans.

Minimally invasive techniques, such as endoscopic endonasal surgery, have also been promising in the treatment of some CVJ malformations, with benefits such as less tissue damage, shorter hospital stay, and faster recovery. However, these techniques require specialized training and equipment and are still being evaluated for their long-term efficacy. The use of autologous bone grafts, such as rib grafts, has also been effective, as evidenced by successful bone fusion and healing at the donor site in children.

Prospective trials should focus on genetic and molecular aspects involved with CVJ anomalies because, according to newer data, the vast majority have a strong genetic involvement. Identifying the exact genetic etiologies will make for earlier and more individualized therapeutic regimens, which can maximize eventual long-term results in these individuals. Studies must be conducted to show the efficacy and reproducibility of the new procedures and technologies implemented within the decade.

## Introduction and background

**Figure 1:**
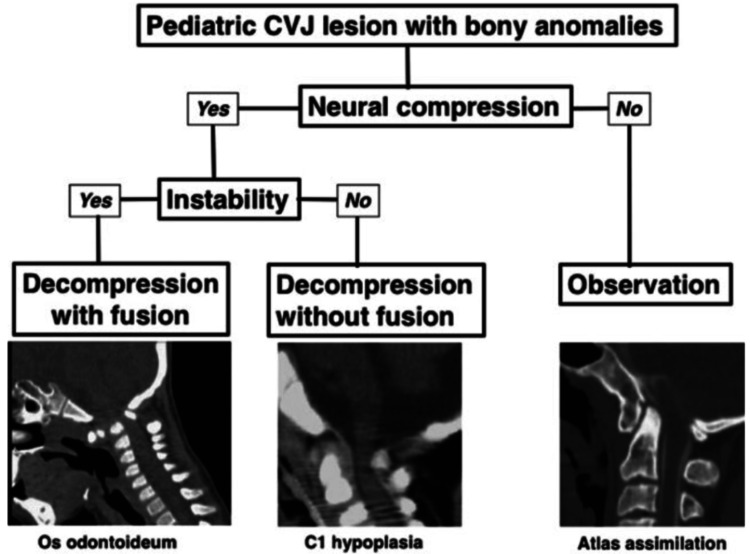
Treatment algorithm for pediatric CVJ bony anomalies based on the practical classification. Image reproduced from Morota (2017) [3] under the Creative Commons (Attribution-NonCommercial-NoDerivatives 4.0 International) license. CVJ = craniovertebral junction

Pediatric craniovertebral junction (CVJ) malformations represent a continuum of congenital and acquired conditions affecting the occipital bone, atlas (C1), axis (C2), and their ligamentous supporting structures [1]. CVJ malformations lead to neurological impairment due to compression of the cervicomedullary junction, vascular insufficiency, or instability. Early diagnosis and prompt surgery are critical to optimize neurological outcomes in affected children [2,3]. Furthermore, a classification and treatment approach for pediatric CVJ bony anomalies, such as basilar invagination and atlantoaxial instability (AAI), are critical for optimizing surgical outcomes (Figure 1) [3]. This review mainly addresses the structural and developmental anomalies of the CVJ. Inflammatory, infectious, and traumatic pathologies were excluded as they were beyond the planned scope.

Chiari malformation type I (CM-I) is one of the most commonly reported CVJ abnormalities in children [4]. A population-based, retrospective cohort study from Kaiser Northern California identified CM-I in 1% of the children undergoing head or spine MRI during the observation period. Median age at diagnosis was 11 years, with headache (55%) and neck pain (12%) being the most common presenting symptoms. Syringomyelia was observed in 12% of the patients on first presentation [5].

CVJ malformation in children is associated with morbidity. Children with trisomy 21, juvenile idiopathic arthritis, upper respiratory infection, and skeletal dysplasias are prone to get affected with CVJ abnormalities [6].

**Figure 2:**
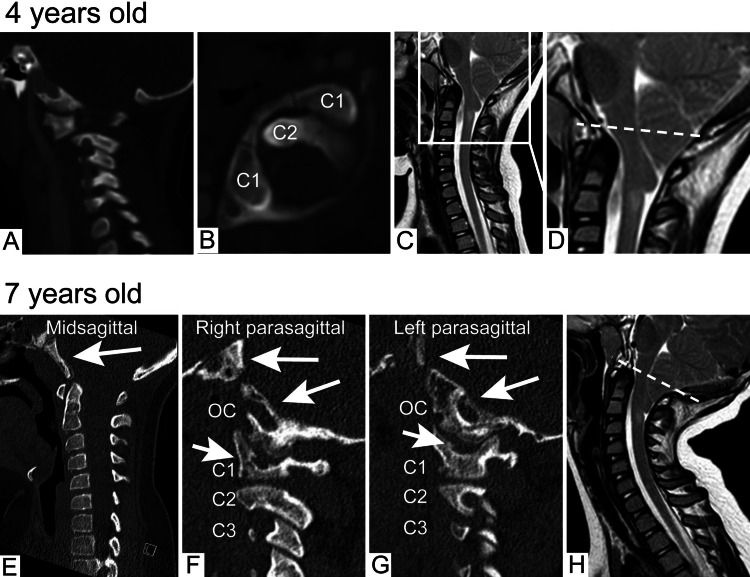
Follow-up imaging when the patient was 10 years old. Midsagittal (A) and parasagittal (B and C) CT demonstrating progressive osteolytic changes in the clivus, OC, and C1 (arrows) now with fusion of the OC to C1. Sagittal MRI (D) of the cervical spine with gadolinium contrast demonstrating worsening of caudal tonsillar descent and progressive bone marrow changes with contrast enhancement involving the CVJ and C1-C4 vertebral bodies (arrow). Note the McRae line (dashed line). Radiograph (E) and bone-window CT images (F) of the skull and skull base (G) demonstrating extensive osteolytic changes of the calvaria, facial bones, and skull. The overall radiological findings were considered diagnostic for GSD. Image reproduced from Syed et al. (2025) [8] under the CC BY-NC-ND 4.0 license. CVJ = craniovertebral junction; GSD = Gorham-Stout disease

The rectus capitis posterior minor plays a significant role in the pathophysiological mechanisms underlying CM-I [7]. The potential presence of denervation damage has been studied. However, future research should be conducted employing methods such as pathology and molecular biology. A four-year-old girl with recurrent symptoms of pharyngitis was initially observed to have cranial settling, basilar impression, and acquired CM-I. Later, when she turned 12 years old, imaging revealed that the girl had developed Gorham-Stout disease, which is a rare group of osteolytic bone disorders (Figure 2) [8].

AAI is another CVJ anomaly of great importance, particularly prevalent in children with Down syndrome. In a study of 404 Down syndrome patients, 14.6% had radiographic evidence of AAI, but only 1.5% were symptomatic and required surgical intervention [9]. In another study, AAI was present in approximately 10-20% of Down syndrome patients, with the disorder primarily being asymptomatic and found radiographically [10].

Technical advances in imaging, particularly MRI and three-dimensional (3D) reconstructed CT, have significantly improved diagnostic accuracy and preoperative planning for CVJ anomalies [11]. Surgical procedures have also undergone transformation, and posterior decompression, occipitocervical fusion, and minimally invasive surgery have provided promising outcomes. Postoperative complications such as failure of hardware and persistent instability remain, with associated challenges in treating these conditions [11].

This literature review aims to make a detailed critique of the pathophysiology, epidemiology, diagnostic innovation, and surgical care of pediatric CVJ anomalies. We also discuss newly emerging trends and future directions in the surgical treatment of these multidisciplinary conditions.

## Review

Epidemiology and pathophysiology

Pediatric CVJ anomalies are a spectrum of congenital and acquired malformations of the occipital bone, atlas (C1), axis (C2), and associated ligamentous structures [1]. Abnormalities can cause neurologic dysfunction due to compression of the cervicomedullary junction, ischemia, or instability. Prompt diagnosis and selective operative intervention are crucial to optimizing neurologic outcomes in affected children [1].

Chiari Malformation Type I

**Figure 3:**
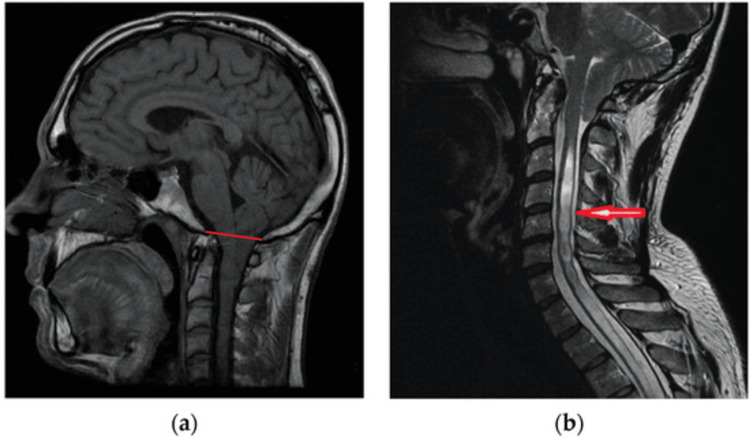
Sagittal MRI (a) Male, 24 years old. CM-I is defined as a displacement of the cerebellar tonsils greater than 5 mm below the basion-opisthion line (red line); (b) Female, 38 years old. CM-I with long-segment syringomyelia (red arrow). Image reproduced from Yan et al. (2024) [12] under the Creative Commons Attribution (CC BY) license. CM-I = Chiari malformation type I

CM-I is formally described as the downward migration of the cerebellar tonsils below the foramen magnum (Figure 3) [12]. In a retrospective cohort study of 5,248 pediatric MRI studies in children under the age of 20 years, CM-I was present in approximately 1% of cases [5]. Headache (55%) and neck pain (12%) were the most common presenting symptoms. Syringomyelia was present in 12% of patients at the time of diagnosis [5].

Atlantoaxial Instability

**Figure 4:**
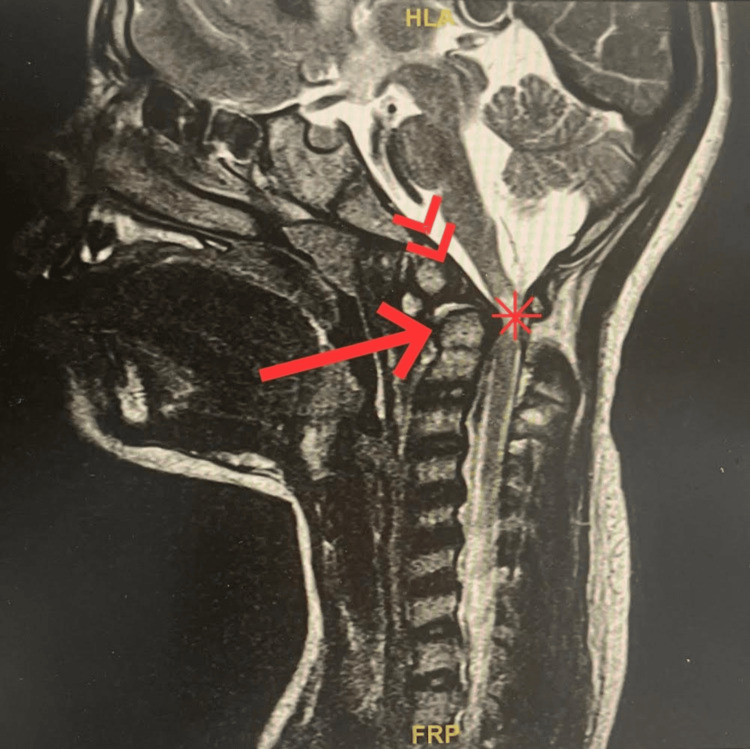
Normal variant os odontoid (double arrow) superimposed with a retroverted odontoid process (arrow) results in the focal compression of the upper cervical cord and subsequent chronic myelopathy (asterisk). Image reproduced from Alfhmi et al. (2023) [13] under the Creative Commons Attribution (CC BY) license.

AAI is a condition defined by excessive movement between the atlas and axis vertebrae. In Down syndrome, AAI is a relatively frequent finding (Figure 4) [13]. A study of 404 Down syndrome patients showed that 14.6% had AAI, with 1.5% presenting with symptomatic neurological deficits [9]. Another study reported that 10% to 30% of Down syndrome patients have radiographic AAI, but symptomatic C1 to C2 instability is present in only about 1% [13].

Grisel’s Syndrome

**Figure 5:**
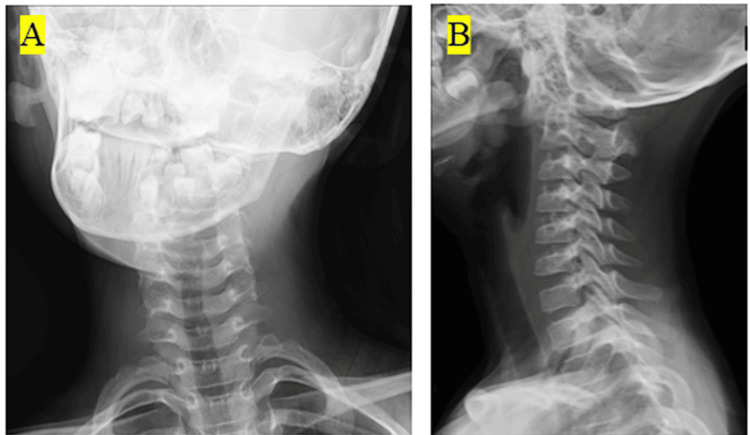
(A) Frontal X-ray image of the cervical spine. An oblique neck position was observed. (B) Lateral radiograph of the cervical spine. Lateral alignment of the cervical spine was intact. Image reproduced from Hashimoto et al. (2024) [14] reproduced under the Creative Commons Attribution (CC BY) license.

Grisel's syndrome is a non-traumatic atlantoaxial subluxation following an upper respiratory infection. It is a rare disorder in children, with an estimated 0.1% of pediatric patients presenting with torticollis and neck pain. The pathophysiology involves laxity of the transverse ligament secondary to inflammation-induced instability. Figure 5 presents an X-ray image of a pediatric patient with Grisel’s syndrome [14].

Pathophysiological Considerations

The pathophysiology of CVJ anomalies is multifactorial, including congenital malformations, developmental abnormalities, and secondary trauma or inflammatory effects. Biomechanical features of the lesions are critical in surgical planning to decide management when instability and compression might coexist, complicating clinical management [1].

Biomechanical Considerations in Pediatric Craniovertebral Junction Anomalies

Pediatric CVJ possesses distinct biomechanical features compared to adults, and these hinge primarily on developmental factors. In children, the cervical spine’s flexion fulcrum is superior at the C2-C3 level compared to adults, where it shifts to the C5-C6 level [15]. This higher fulcrum in children is a direct consequence of a proportionally greater head-to-body size ratio and immature cervical musculature, rendering them more vulnerable to upper cervical spine trauma. In addition, pediatric vertebral facets are more horizontally oriented, presenting less resistance to anterior translation and causing greater mobility at the CVJ. Such anatomical and biomechanical differences necessitate age specificity in the diagnosis and treatment of CVJ anomalies [15].

Diagnostic challenges in pediatric craniovertebral junction anomalies

Pediatric CVJ anomalies are diagnosed with special difficulties involving overlapping symptoms of other diseases in children and the fragility of clinical presentations. Common symptoms such as neck ache, torticollis, and gait disturbance are easily attributed to less harmful conditions, leading to late diagnosis [16]. Furthermore, the usual radiographic measurements employed in adults for the assessment of CVJ instability are not directly extrapolated to children due to ongoing skeletal development. The typical adult normal atlantodental interval may not be equivalent in children, for instance, making it impossible to assess AAI. Advanced imaging modalities, such as dynamic CT and MRI, are important for accurate assessment, but their interpretation requires knowledge of pediatric spinal anatomy [16].

Diagnostic modalities

A recent editorial has emphasized the importance of neuroimaging in the diagnosis of neurological conditions [17]. Proper diagnosis of CVJ anomalies in children is crucial to allow appropriate management and surgical planning. Various imaging modalities, including MRI, CT, and dynamic imaging modalities, play a crucial role in the evaluation of these complex anomalies [6].

Role of MRI, CT, and Dynamic Imaging in Diagnosis

MRI remains the standard tool for assessing soft tissue structures in the CVJ, including the spinal cord, brainstem, ligaments, and cerebrospinal fluid compartments [18]. It is superior for depicting contrast within and between these tissues and detecting complications such as Chiari malformation, syringomyelia, and ligament injury. MRI can be particularly useful in grading neural compression, along with its attendant signal change, in the spinal cord [18].

CT imaging is superior for the visualization of bony anatomy and invaluable in the identification of osseous abnormalities such as basilar invagination, atlantoaxial dislocation, and congenital vertebral fusions. Multiplanar and 3D reconstructions enhance assessment of complex bony anatomy and are essential in preoperative planning [6].

Dynamic imaging, including flexion-extension radiographs and dynamic CT scans, is extremely valuable to evaluate CVJ instability. Dynamic CT, in particular, has been shown to be beneficial in establishing atlantoaxial rotatory fixation (AARF) in children with chronic torticollis that does not respond to conservative management. Early diagnosis and treatment of AARF are crucial to prevent its progression to chronic fixation [6].

Emerging Imaging Techniques and Their Clinical Relevance

**Figure 6:**
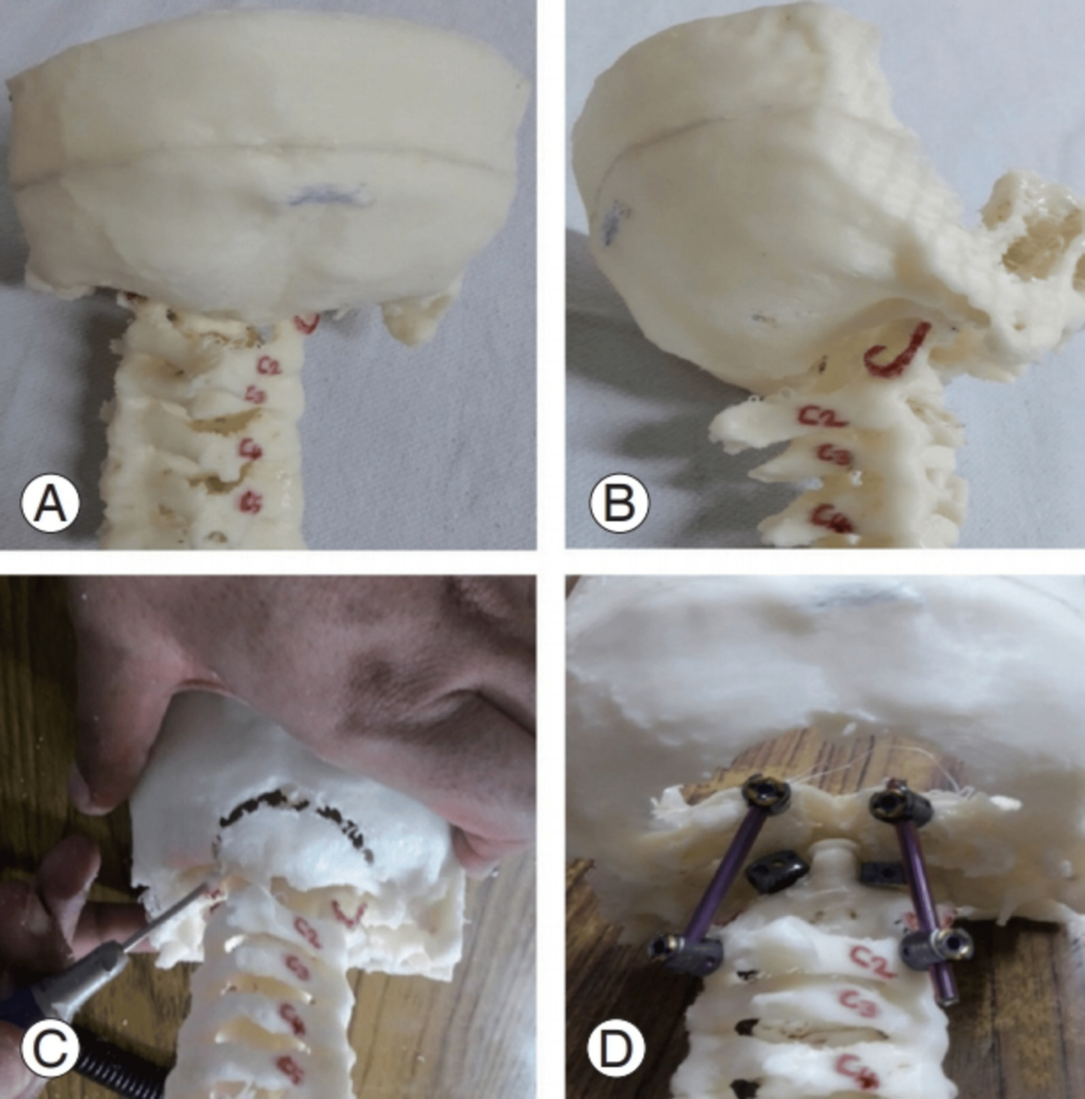
(A) A 3D-printed model of a patient with occipitalized atlas. (B) Lateral view of the model. (C) Practice using the model. (D) Model with occiput-C2 screws. Image reproduced from Agarwal et al. (2020) [19] under the Creative Commons Attribution Non-Commercial License.

Advances in imaging software have made it possible to create 3D models from CT and MRI scans for improved visualization of CVJ anomalies (Figure 6) [19]. The models aid in preoperative planning by providing detailed anatomical information, such as vertebral artery course and bony structure spatial relationships. Such careful visualization is particularly helpful in complex cases for surgical management [20].

The use of intraoperative CT-based navigation systems has improved the accuracy of CVJ surgery. They enable imaging in real time, enabling precise anatomical landmark localization and secure instrumentation placement. This technology has been associated with reduced surgical complications and improved outcomes in pediatric CVJ surgery [11].

Surgical management strategies

Pediatric CVJ anomalies are indicated for surgical intervention in cases of symptomatic instability, neural compression, or progressive deformity. The choice of surgical approach depends on the underlying pathology, anatomical factors, and the presence of associated comorbid conditions [3].

Indications for Surgical Intervention

In children with Down syndrome, symptomatic CVJ instability is a common reason for surgery. A systematic review of 38 reports determined that 81% of patients underwent surgery due to symptomatic, radiologically confirmed CVJ instability. Myelopathy (30%), weakness (25%), gait abnormality (24%), torticollis (15%), and neck pain (14%) were the most frequent presenting symptoms [21].

Overview of Surgical Techniques

Posterior methods are the mainstay for the treatment of CVJ anomalies. In a series of 78 patients with CVJ deformities and torticollis, posterior correction and fusion were found to effectively restore the anatomical alignment. The mean operating time was 115.6 ± 12.8 minutes, and the mean blood loss was 170.8 ± 26.3 mL [22].

Occipitocervical fusion is employed in cases of the occipital and upper cervical spine. According to previous research, occipitocervical fusion via screw and wire systems is an effective and useful method for the treatment of CVJ instability with excellent fixation and cervical decompressive operations [2].

Minimally invasive techniques, such as endoscopic endonasal odontoidectomy, have advantages in some cases. A case series of seven children with combined posterior occipitocervical decompression and endoscopic endonasal odontoidectomy reported that the mean day of postoperative feed initiation was postoperative day 1.0, evidencing an excellent recovery profile [23].

Posterior Care and Complication Management

Postoperative care involves close monitoring for postoperative complications such as failure of hardware, infection, and neurological worsening. In a consecutive series of 22 patients with posterior occipitocervical fusion for basilar invagination, 80.95% of patients had neurological improvement by at least one grade. However, one (4.54%) patient died in the postoperative period due to respiratory insufficiency, which emphasizes the importance of close postoperative monitoring [24].

Outcomes and prognostic factors

Surgical treatment of pediatric CVJ malformations has been linked with optimistic outcomes, particularly when both timely and adequately performed. However, the prognosis is influenced by a wide range of factors, including the pathology, surgical technique, and patient-specific factors.

In a prospective analysis of 34 patients with CVJ pathologies, surgical interventions such as occipitocervical fusion and C1-2 fusion were linked with dramatic improvements. The preoperative mean atlanto-dens interval decreased from 6.6 ± 2.3 mm to 4.2 ± 0.6 mm postoperatively. Similarly, the space available for the cord also decreased from 8.3 ± 2.9 mm to 17.2 ± 1.6 mm, and the clivus canal angle also increased from 130.2 ± 15.3° to 143.3 ± 8.3°, indicating successful decompression and alignment. The patients also demonstrated statistically significant improvement in neurological function based on the American Spinal Injury Association impairment scale and the modified Japanese Orthopedic Association score [25].

In a systematic review involving 38 studies, 81% of pediatric Down syndrome patients treated surgically for CVJ instability were found to have symptomatic, radiologically confirmed instability. The most common presenting initial symptoms were myelopathy (30%), weakness (25%), gait abnormality (24%), torticollis (15%), and neck pain (14%). The most common surgical procedures performed were posterior occipitocervical or atlantoaxial instrumented fusion, each in 57% and 44% of patients, respectively. The overall surgical mortality was 3%, and the complication rates were 36% [21].

A pediatric study in which posterior fossa reconstruction was performed on patients with Chiari malformations showed that, at the one-year follow-up, 91.9% of children had good results, 4.5% had fair results, and 3.6% had bad results. There were zero neurological deterioration and deaths in the group, yet 30% of patients developed at least one postoperative adverse event, of which aseptic meningitis was the most frequent (22.7%) [26].

The use of autologous rib grafts in craniocervical junction fusion surgery has also yielded good results. In a retrospective study of 10 children, all of whom had good clinical results, bone union was confirmed by CT at three to six months post-operation. No neurological or donor site complications were observed during the follow-up of 8 to 24 months [27].

The determinants of outcome and recovery include the comorbidity status, the severity of the anomaly, and the timing of intervention [2]. Early diagnosis followed by treatment correlates with better neurological function and a lower complication rate, whereas delayed intervention leads to intractable neurological damage and an augmented risk of operation [28].

Emerging trends and future directions

The progress in the management of pediatric CVJ abnormalities has been fueled by developments in surgical techniques, imaging technology, and genetics. Such developments will continue to enhance diagnostic accuracy, surgical precision, and outcomes for patients [3].

Innovations in Surgical Techniques and Technology

Intraoperative navigation system integration has improved both the effectiveness and safety of CVJ surgeries significantly. One study highlighted that intraoperative CT-based navigation through the form of congenital CVJ anomalies provided real-time imaging, facilitating precise screw placement and reducing neurovascular complications [11]. This type of technique has been associated with improved postoperative results as well as reduced operating times [11].

3D printing technology has been recognized as a valuable adjunct to preoperative planning in planning complex CVJ operations [29]. Patient-specific anatomical models can be created to visualize the individual anatomical variations better and plan accordingly. Personalization has been shown to enhance the accuracy of surgery and reduce intraoperative complications [29].

**Figure 7:**
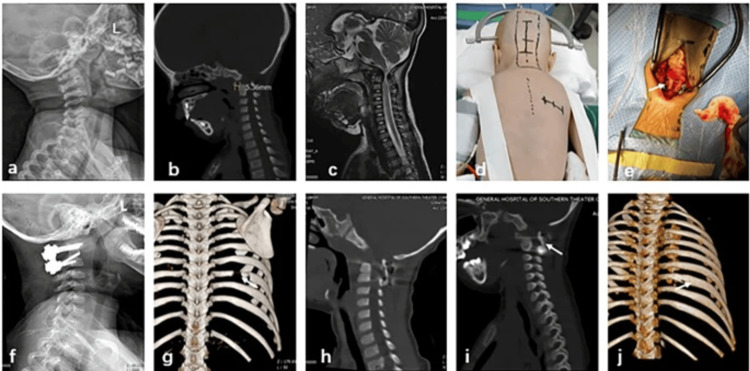
An atlantoaxial dislocation was diagnosed in a two-year-old girl. (a-c) Preoperative MRI, CT, and X-rays demonstrating spinal canal stenosis, atlantoaxial dislocation, and atlantodental interval widening. (d) Marking the location of the right-sided oblique rib incision and cervical incision. (e) Autologous ribs are used intraoperatively for structural bone grafting. (f) Postoperative X-ray demonstrates that the C1-C2 internal fixation is positioned correctly. (g) A week following surgery, a chest CT 3D reconstruction reveals a defect in the right eighth rib. (h) A week following surgery, a cervical sagittal CT scan demonstrates that the rib bone transplant was positioned correctly. (i) Three months following surgery, a follow-up cervical spine CT scan reveals bone fusion at the graft site. (j) Three months following surgery, a chest CT 3D reconstruction demonstrates full healing of the donor site’s rib defect. Image reproduced from Deng et al. (2024) [27] under the Creative Commons Attribution 4.0 International License.

The introduction of minimally invasive surgical techniques, such as endoscopic endonasal procedures, has increased the potential for surgical treatment of CVJ anomalies. These operations have the advantages of less tissue disruption, reduced hospital stays, and quicker recovery times [30]. Their application, however, requires additional training and equipment, and their long-term outcomes are still being evaluated. For example, in a case involving a two-year-old female with atlantoaxial dislocation, intraoperative navigation and autologous rib grafting demonstrated effective stabilization and bone fusion, as evidenced by postoperative imaging at three months showing complete regeneration of the rib defect and successful C1-C2 fixation (Figure 7) [27].

Potential for Genetic and Molecular Research in Understanding Craniovertebral Junction Anomalies

Studies over the past decade have highlighted the genetic nature of certain CVJ abnormalities. For instance, mutations involving the *KMT2A* gene have been identified with Wiedemann-Steiner syndrome, a syndrome characterized by the presence of facial features, mental retardation, and skeletal defect, including deformity of the CVJ. In 11 patients with a mutation in the *KMT2A* gene reported in a study, 10 presented with anomalies of the CVJ, pointing to the contributing role of genes in CVJ pathology [31].

Similarly, developmental osseous CVJ malformations, such as atlantoaxial dislocation and basilar invagination, have been associated with genetic effects on joint anatomy and ligamentous morphology [32]. Clarification of these genetic effects is essential for early diagnosis and the institution of particular therapies.

The investigation of molecular mechanisms implicated in bone formation and joint development continues to shed light on the etiology of CVJ anomalies. Advances in genetic and molecular studies hold the potential for the discovery of new therapeutic targets and the creation of personalized treatment approaches. Furthermore, further research into the age-related effects of the brain can provide more insights into the treatment and diagnosis of CVJ anomalies [33,34].

## Conclusions

Pediatric CVJ anomalies are a complex and heterogeneous group of congenital and acquired conditions that pose a significant challenge to diagnosis and management. Despite advances in imaging technology, surgical techniques, and personalized medicine, the treatment of these anomalies is challenging due to the unique anatomical and biomechanical characteristics of the pediatric spine. Emerging technologies such as intraoperative CT-based navigation, 3D printing, and minimally invasive surgery have improved the outcome by optimizing the accuracy of the surgery and reducing complications. However, a large proportion of existing evidence is derived from case reports and small series, imposing limitations on drawing reliable conclusions about the comparative efficacy of interventions. The long-term efficacy of these technologies must be proven, and the genetic and molecular underpinnings of these anomalies are not yet well elucidated. Future research should concentrate on linking genetic findings to clinical practice to develop more effective, targeted treatment plans, ultimately enhancing the quality of life in these children.
